# Critical Aortic Stenosis With Rapid Multiorgan Decompensation Reversed by Emergency TAVR

**DOI:** 10.1016/j.jaccas.2026.109639

**Published:** 2026-08-05

**Authors:** David G. Gent, Majid Jafar, Marwa Daghem, Kully Sandhu, Warkaa Shamkhani

**Affiliations:** a Cardiology Department, Liverpool Heart and Chest Hospital NHS Foundation Trust, Liverpool, United Kingdom; b Internal Medicine Department, Saudi-German Hospital, Dubai, UAE; c Cardiovascular Research Group, Keele University, Keele, United Kingdom

**Keywords:** aortic stenosis, bicuspid aortic valve, cardiogenic shock, multiorgan failure, pulmonary edema, transcatheter aortic valve replacement

## Abstract

**Background:**

Critical aortic stenosis can deteriorate abruptly from compensated disease to low-output shock and multiorgan dysfunction. We describe rapid multiorgan decompensation from severe bicuspid aortic stenosis with recovery following emergency transcatheter aortic valve replacement (TAVR).

**Case Summary:**

A previously active 69-year-old man with severe bicuspid aortic stenosis re-presented to the emergency department with pulmonary edema and rapidly progressive breathlessness. Although systolic blood pressure was preserved, he had severe hypoperfusion with lactate 8.6 mmol/L, alanine aminotransferase 5,379 IU/L, international normalized ratio 2.8, creatinine 189 μmol/L, and bilirubin 48 μmol/L. Repeat echocardiography demonstrated new biventricular dysfunction, functional mitral regurgitation, and a high probability of pulmonary hypertension. He underwent emergency transfemoral TAVR, which reduced the gradient from 129 to <10 mm Hg. The biochemical abnormalities improved within 48 hours, and he was discharged well 8 days later.

**Discussion:**

This case illustrates rapid, reversible multiorgan decompensation caused by critical aortic stenosis.

**Take-Home Messages:**

Deteriorating critical aortic stenosis should prompt urgent reassessment and expedited intervention. Emergency TAVR can restore forward flow and facilitate rapid physiologic recovery.

## Visual Summary

**Figure:**
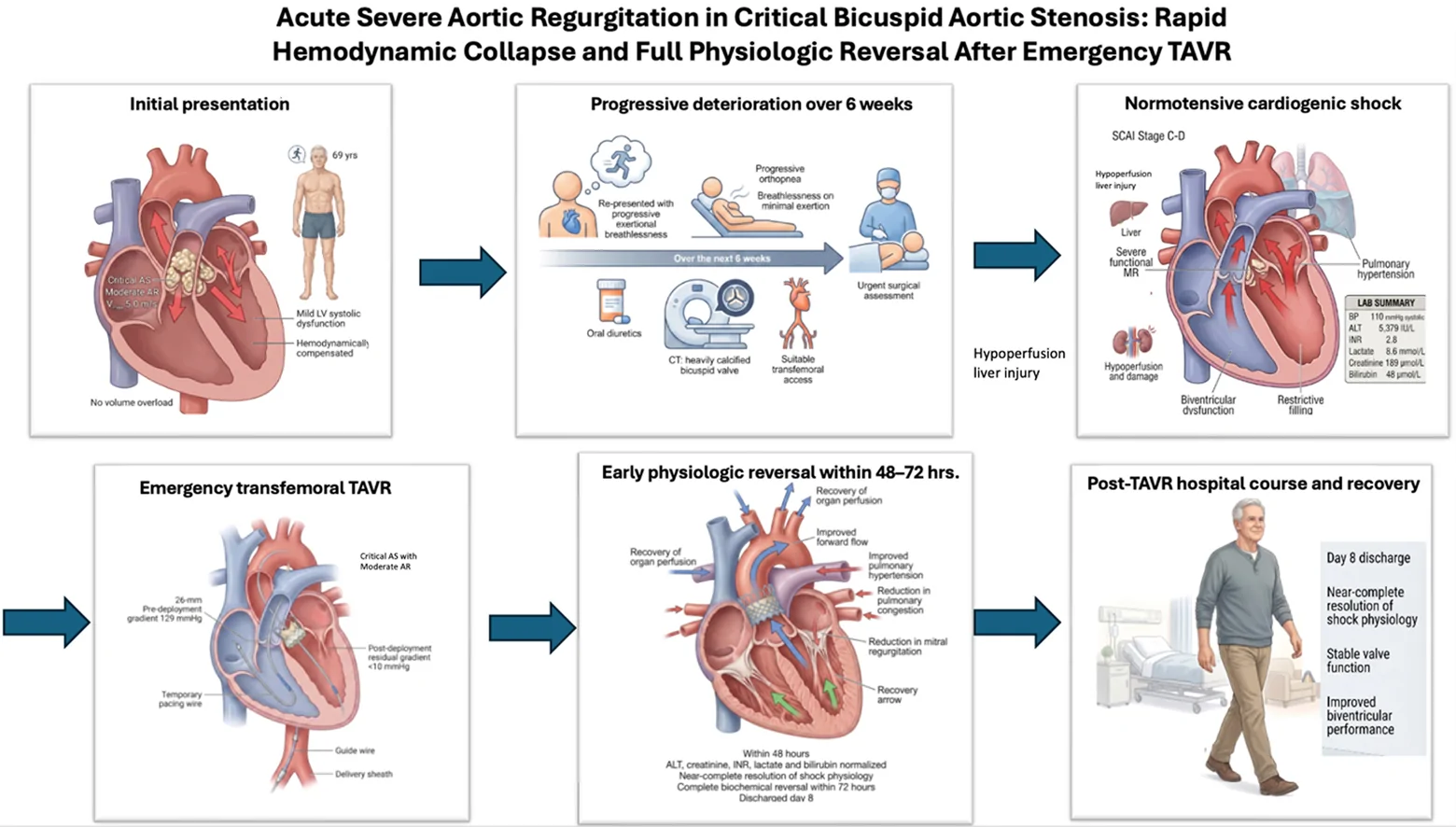

## History of Presentation

A previously active 69-year-old man with no comorbidities attended the emergency department of the local hospital with progressive exertional breathlessness. Transthoracic echocardiography was performed, revealing critical aortic stenosis (AS) (Vmax: 5.0 m/s), moderate aortic regurgitation (AR), and mild left ventricular (LV) systolic dysfunction (Figure 1, Videos 1 and 2). The patient was hemodynamically compensated with no signs of volume overload and was referred to cardiology for urgent outpatient follow-up. He remained physically active, attending the gym 3 times weekly. Over the next 6 weeks (Figure 2), he re-presented to the emergency department with progressive exertional breathlessness and was treated with oral diuretics pending outpatient cardiology review. A computed tomography (CT) scan of his aortic valve and aorta was performed to help guide planned intervention. The CT showed a heavily calcified bicuspid valve with suitable transfemoral access and no significant coronary artery disease (Figure 3). Given the progressive symptoms, he was referred for urgent surgical outpatient assessment. He deteriorated over the following week, with progressive orthopnea and breathlessness on minimal exertion.

**Take-Home Messages**

• Clinical deterioration in severe aortic stenosis should prompt urgent repeat echocardiography and accelerated valve-team reassessment, even when recent imaging has been performed.
• In selected patients with valve-driven shock and suitable anatomy, emergency TAVR can rapidly restore forward flow and reverse multiorgan dysfunction.

**Figure 1:**
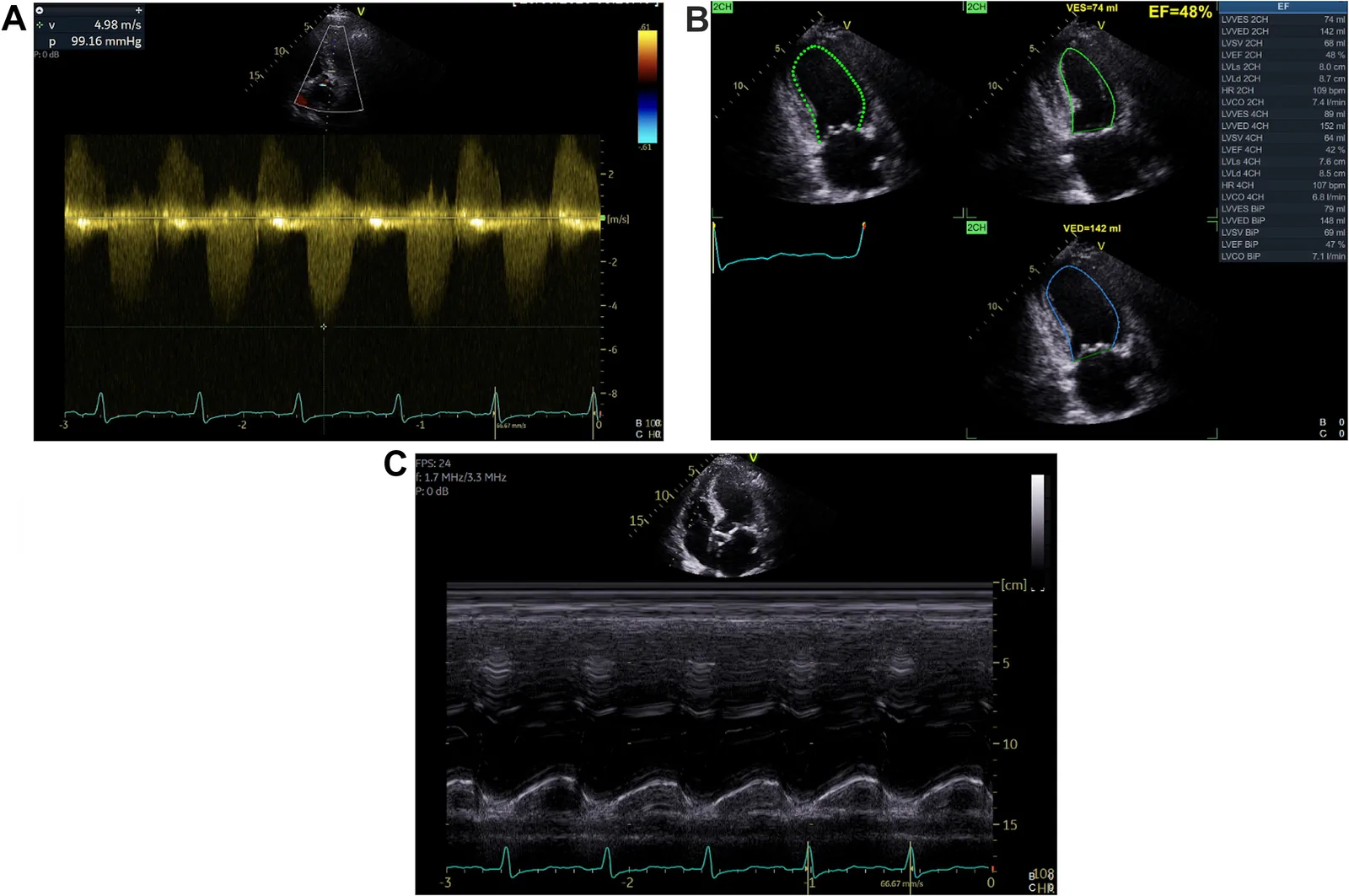
Baseline Echocardiogram Baseline echocardiography at the initial presentation demonstrated (A) severe aortic stenosis with moderate aortic regurgitation, (B) normal left ventricle size with moderately impaired systolic function (LVEF: 48%), (C) preserved right ventricular systolic function (TAPSE: 18 mm). LVEF = left ventricular ejection fraction; TAPSE = tricuspid annular plane systolic excursion.

**Figure 2:**
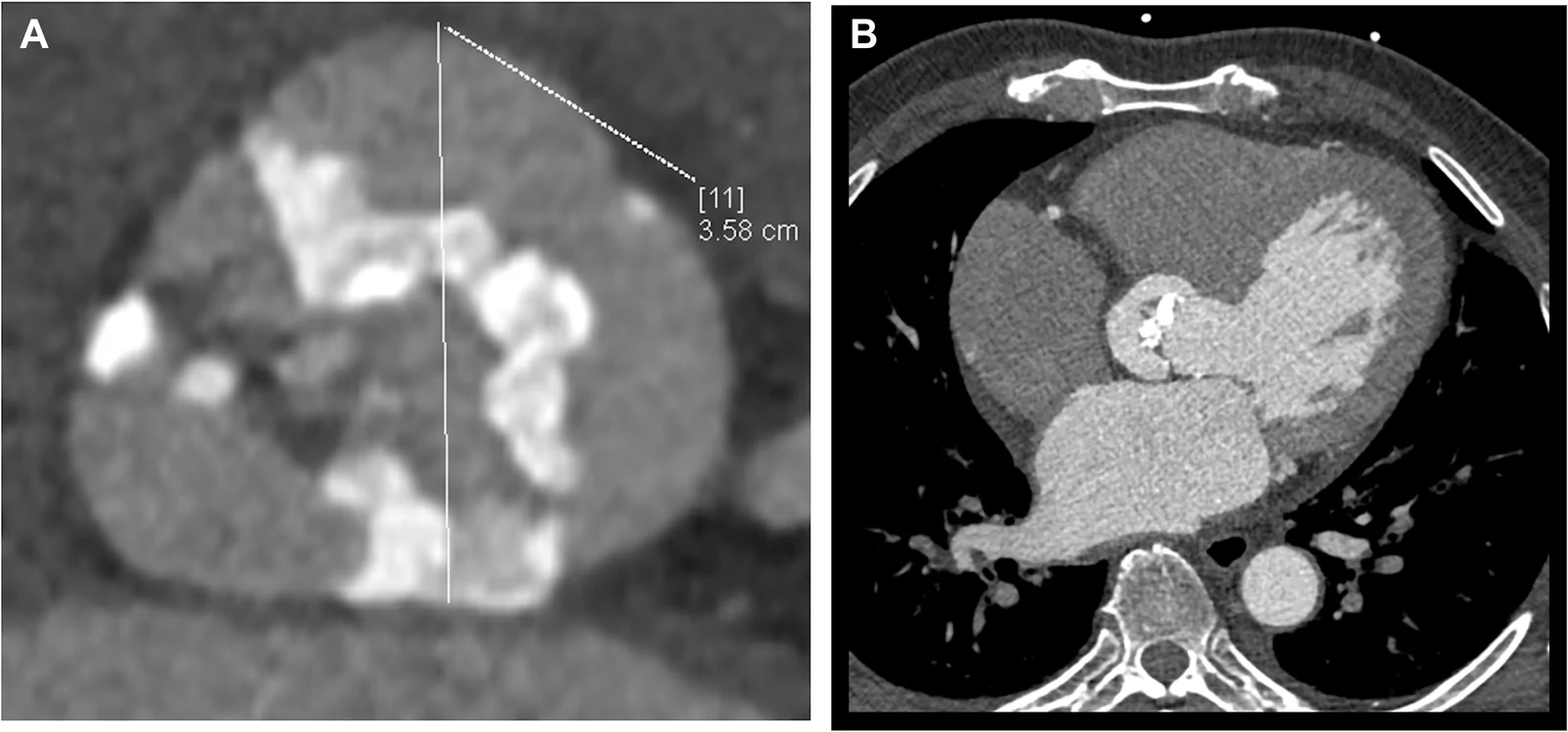
Aortic Valve and Coronary Computed Tomography Scan Cardiac-gated computed tomography scan showing (A) bicuspid aortic valve with severe calcification and (B) aortic valve calcification with normal left ventricular size and concentric hypertrophy (diastolic phase, 30% of the cardiac cycle).

**Figure 3:**
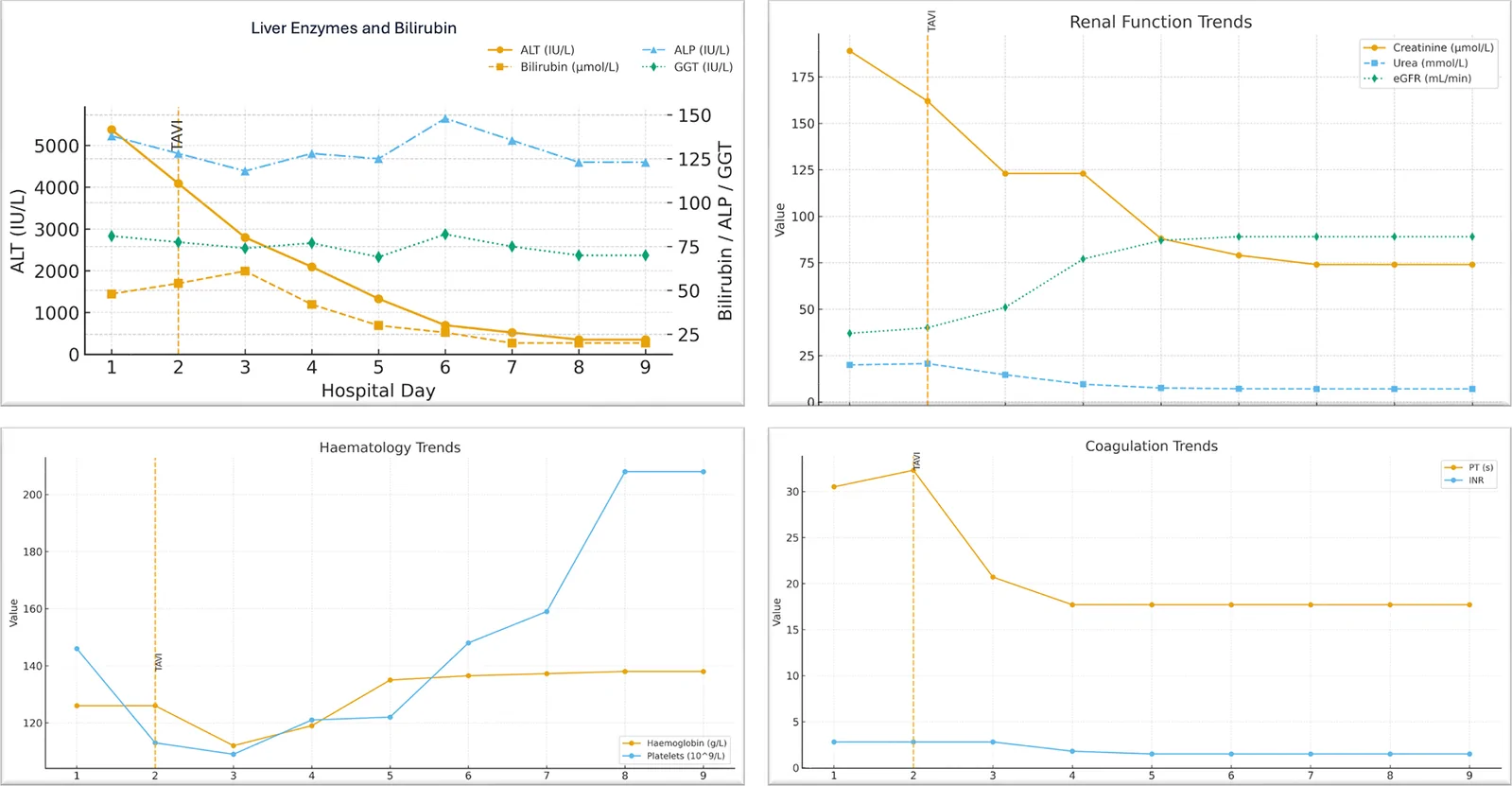
Laboratory Trajectory (Liver Function Tests, Renal, Coagulation, Hematology) Serial blood tests showed rapid improvement in liver function, renal function, coagulation profile, lactate, and hematologic parameters after emergency TAVR. ALT, creatinine, INR, lactate, and bilirubin normalized within 48 hours, supporting reversible multiorgan hypoperfusion from valve-driven low-output shock rather than primary hepatic or renal pathology. ALT = alanine aminotransferase; ALP = alkaline phosphatase; eGFR = estimated glomerular filtration rate; GGT = gamma-glutamyl transferase; INR = international normalized ratio; PT = prothrombin time; TAVR = transcatheter aortic valve replacement.

He then presented in extremis to the emergency department, 6 weeks after his initial presentation, with normotensive cardiogenic shock (SCAI [Society for Cardiovascular Angiography and Interventions] stage C-D). His blood pressure was stable at 110 mm Hg systolic with profound biochemical evidence of hypoperfusion: alanine aminotransferase (ALT) 5,379 IU/L, international normalized ratio (INR) 2.8, lactate 8.6 mmol/L, creatinine 189 μmol/L, and bilirubin 48 μmol/L. The working diagnosis was ischemic hepatitis, cardiogenic acute kidney injury, and global low-output physiology (Figure 4). He was transferred emergently to the regional transcatheter aortic valve replacement (TAVR) center, where he was stabilized with intravenous furosemide infusion, vitamin K, and fresh-frozen plasma.

**Figure 4:**
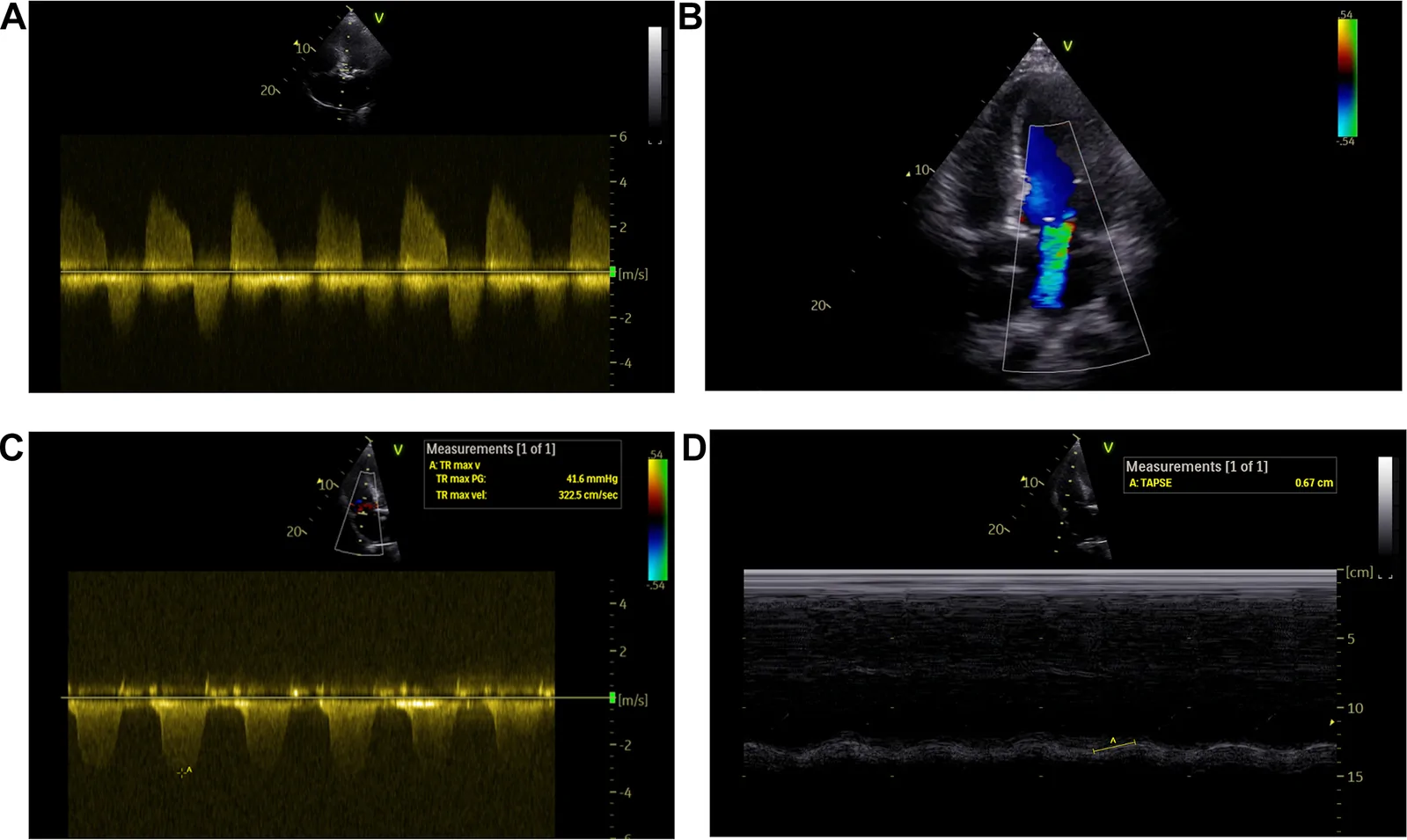
Decompensated Critical Aortic Stenosis With Biventricular Dysfunction Transthoracic echocardiography at clinical deterioration demonstrated (A) a transthorasic Doppler profile within the moderate aortic stenosis range, (B) functional mitral regurgitation, (C) tricuspid regurgitation with a peak velocity of 3.23 m/s and a peak gradient of 41.6 mmHg consistent with an elevated echocardiographic probability of pulmonary hypertension, and (D) severely impaired right venticular longitudinal systolic function, with a tricuspid annular plane systolic excursion of 0.67 cm.

Repeat echocardiography (Figure 5, Video 3) showed new severe biventricular dysfunction with restrictive filling, moderate to severe functional mitral regurgitation (MR), biatrial dilatation, and high probability of pulmonary hypertension (pulmonary arterial systolic pressure ≥42 mm Hg, dilated right atrium [24 cm^2^], and dilated inferior vena cava [2.3 cm] with no respiratory variation). The moderate AR was unchanged from 6 weeks previously.

**Figure 5:**
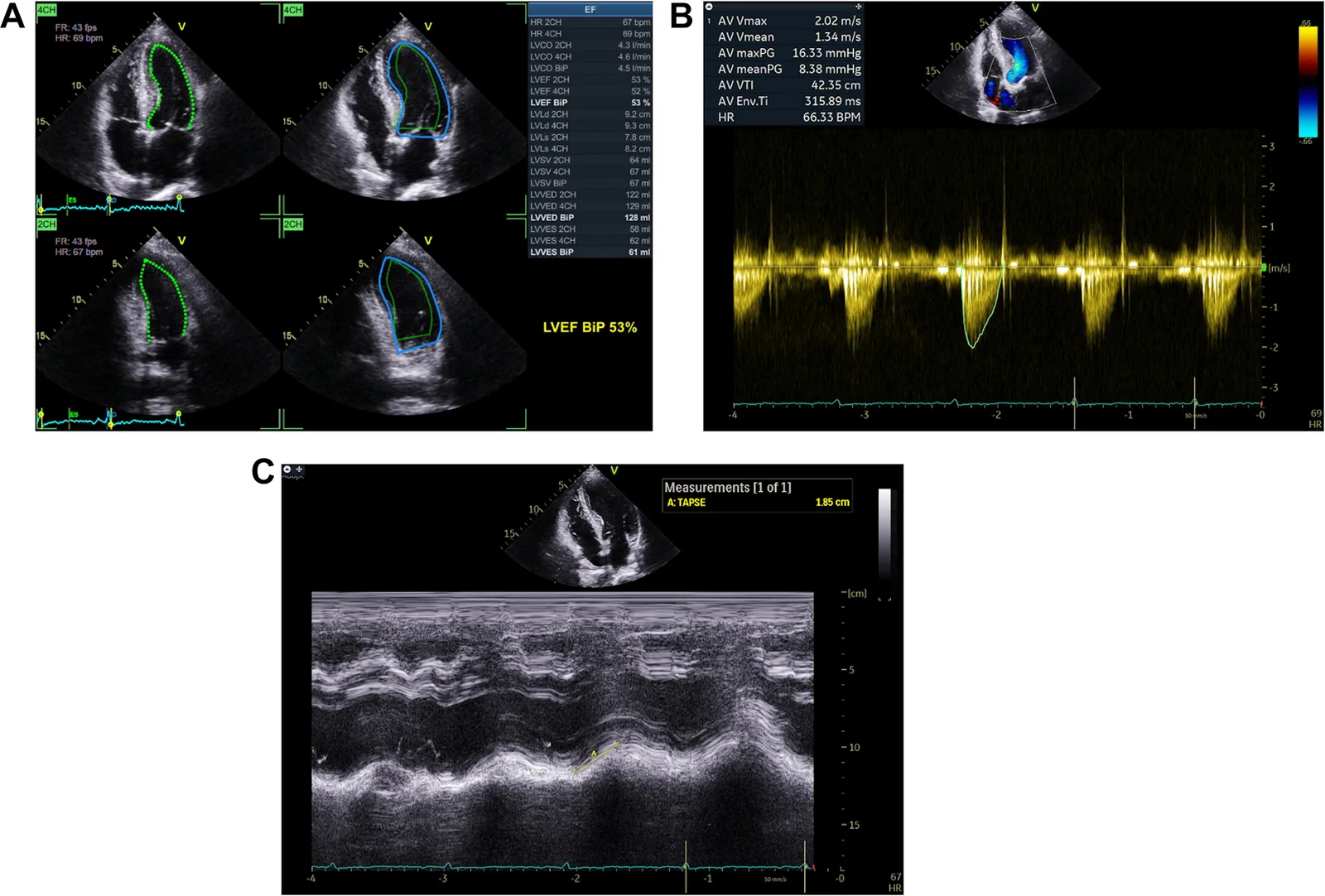
Transthoracic Echocardiography 2 Months After TAVR Post-TAVR echocardiography showed (A) improved left ventricular systolic function, (B) reduced aortic valve gradient with resolution of aortic regurgitation, and (C) improved right ventricular systolic function, with significant increase in TAPSE (19 mm). There was no detectable tricuspid regurgitation and low echocardiographic probability of pulmonary hypertension. TAPSE = tricuspid annular plane systolic excursion; TAVR = transcatheter aortic valve replacement.

## Past Medical History

The patient had no significant past medical history and was previously physically active.

## Differential Diagnosis

The differential diagnosis included acute severe AR, acute coronary syndrome, arrhythmia, infective endocarditis, and aortic dissection, The index of suspicion for endocarditis and dissection was low, and there were no supportive clinical features, vegetations, abscess formation, or aortic root dissection present on cardiac imaging.

## Management

After stabilization, the patient underwent emergency transfemoral TAVR with anesthetic support and pre-emptive temporary pacing. A 26-mm Sapien 3 valve (Edwards Lifesciences) was selected, with +1 mL overfill planned to ensure adequate expansion.

Right radial access was used for monitoring, and right femoral access was obtained under ultrasound guidance with 2 ProGlides (Abbott). After crossing the valve, a 22-mm balloon predilatation was performed. A 26-mm Sapien 3 Ultra valve (Edwards Lifesciences) was deployed, with +1 mL added. The predeployment gradient was 129 mm Hg; the postdeployment residual gradient was <10 mm Hg with an AR index of 38, indicating excellent relief of obstruction and no significant residual AR. Angiography confirmed no paravalvular leak or vascular complications. The pacing wire and femoral sheath were removed with successful closure. The patient was transferred to the cardiac care unit on intravenous furosemide and enoximone, with plans for routine blood tests, electrocardiography, and echocardiography the following morning.

## Outcome and Follow-Up

Within 48 hours, the ALT, creatinine, INR, lactate, and bilirubin normalized (Figure 4). The biventricular function started to improve, with reduction in MR and pulmonary hypertension. He was discharged on day 8 after TAVR, with near-complete resolution of shock physiology. A follow-up echocardiogram showed stable valve function and improved biventricular performance (Video 4).

## Discussion

Cardiogenic shock in the context of severe AS carries substantial mortality, and urgent valve intervention remains the only therapy with survival benefit. Although the safety and success of urgent TAVR to treat patients with severe aortic stenosis and cardiogenic shock has been described previously, it remains under-reported.^1^, ^2^, ^3^ Contemporary registry and meta-analysis consistently demonstrate that emergent TAVR, although higher risk than elective procedures, is superior to medical management alone in cardiogenic shock.^4^, ^5^, ^6^, ^7^ This case supports this evidence, by demonstrating complete reversal of multiorgan dysfunction, including ischemic hepatitis, coagulopathy, and acute kidney injury, within 72 hours of TAVR. Importantly, the patient's initial echocardiogram 6 weeks earlier showed only mild to moderate AR, preserved right ventricular function (RV), mild LV systolic dysfunction, no pulmonary hypertension, and normal mitral and tricuspid valve function. The subsequent development of severe biventricular dysfunction, functional MR and tricuspid regurgitation, pulmonary hypertension, and multiorgan shock thus represents a physiological inflection point rather than the expected disease trajectory. This underscores how rapidly biventricular dysfunction with a global low-output state can develop in the setting of critical AS, prompting urgent reassessment when symptoms escalate.

In critical AS, the LV operates at the limits of compensatory reserve. The sudden decompensation of LV systolic function in a noncompliant, hypertrophied LV results in a rapid elevation in afterload and LV end-diastolic pressure, acute functional MR, and postcapillary pulmonary hypertension, with increased RV afterload, RV dilatation and dysfunction, and severe tricuspid regurgitation, as demonstrated in this case.

The significant biochemical derangements, ALT >5,000 IU/L, INR 2.8, lactate 8.6 mmol/L, and creatinine 189 μmol/L, are characteristic of global low-output hypoperfusion and fulfill criteria for stage C-D *normotensive* cardiogenic shock, despite a preserved blood pressure of 110/50 mm Hg. In contemporary shock classification, elevated lactate and end-organ dysfunction define true cardiogenic shock, whereas hypotension is not required. The rapid and near-complete normalization of hepatic, renal, and coagulation parameters within 48 to 72 hours after restoration of forward flow with TAVR strongly supports a purely hemodynamic mechanism. This dramatic normalization highlights an important concept: multiorgan dysfunction caused by valvular obstruction can be fully reversible when forward flow is promptly restored.

This case underscores another important learning point: In patients with known severe AS, presentations with shortness of breath or escalating symptoms should prompt urgent reassessment with repeat echocardiography, irrespective of how recently the prior study was performed. Acute deterioration in ventricular function may occur abruptly in a nonlinear fashion and significantly accelerate hemodynamic deterioration.

Finally, this case reinforces the expanding role of salvage TAVR in hemodynamically unstable patients with anatomy suitable for transfemoral access. Although cardiogenic shock has historically been associated with poor outcomes after TAVR, emerging evidence, and the complete recovery demonstrated here, supports its use when the underlying mechanism is valve-driven and rapidly reversible.

## Conclusions

Rapid progression of biventricular dysfunction in critical aortic stenosis can precipitate sudden multivalvular failure and fulminant multiorgan collapse. In selected patients with valve-driven shock and suitable anatomy, emergency TAVR can rapidly reverse severe shock physiology when performed promptly. This case underscores the importance of early re-evaluation and accelerated treatment pathways for patients with deteriorating aortic stenosis.

**Visual summary:**
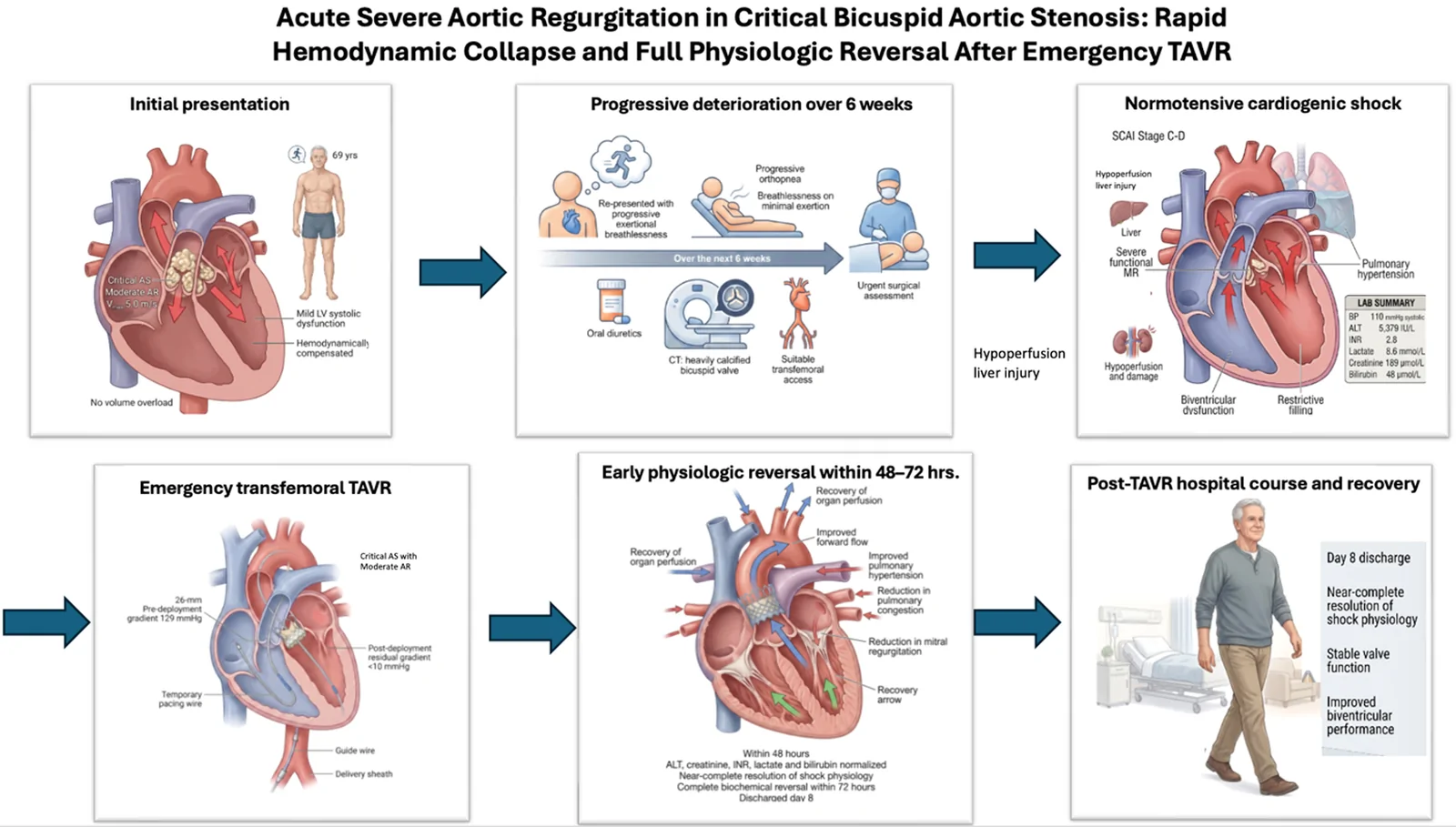
Emergency TAVR for Critical Bicuspid Aortic Stenosis With Multiorgan Shock A Previously active 69-year-old man with critical aortic stenosis, moderate aortic regurgitation, and mild left ventricular systolic dysfunction initially remained hemodynamically compensated. Over 6 weeks, he developed progressive breathlessness and later presented in extremis with normotensive cardiogenic shock and severe biochemical hypoperfusion. Repeat echocardiography demonstrated new biventricular dysfunction, restrictive filling, functional mitral regurgitation, and high probability of pulmonary hypertension. Emergency transfemoral TAVR restored forward flow, reducing the transvalvular gradient from 129 to <10 mm Hg, with no significant residual aortic regurgitation. Within 48 hours, hepatic, renal, coagulation, and lactate abnormalities normalized. ALT = alanine aminotransferase; AR = aortic regurgitation; AS = aortic stenosis; BP = blood pressure; CT = computed tomography; INR = international normalized ratio; LV = left ventricular; MR = mitral regurgitation; SCAI = Society for Cardiovascular Angiography and Interventions; TAVR = transcatheter aortic valve replacement.

## Funding Support and Author Disclosures

The article publication charges were funded by Edwards Lifesciences. Edwards Lifesciences had no role in the preparation, content, review, or approval of the manscript. The authors have reported that they have no relationships relevant to the contents of this paper to disclose.

## Appendix

Video 1 Parasternal Short-Axis View Showing the Severely Stenotic Bicuspid Aortic Valve With Right and Left Coronary Cusp Fusion (Sievers Type 1)

Video 2 Apical 5-Chamber View Showing Mildly Impaired Biventricular Function and Mild to Moderate AR 6 Weeks Before Emergency Admission

Video 3 Apical 4-Chamber View of the Emergency Admission Echocardiogram Showing New Biventricular Severe Dysfunction and Atrial Dilatation

Video 4 Apical 5-Chamber View Showing Well-Seated TAVR With Resolution of Valve Incompetence and Improved Biventricular Function
